# Virtual Money and Emotional Reality: A Qualitative Analysis of Symbolic Issues in a Stock Market Simulation

**DOI:** 10.3390/bs16081440

**Published:** 2026-08-20

**Authors:** Alain Finet, Kevin Kristoforidis, Julie Laznicka

**Affiliations:** Financial Management Department, University of Mons, 7000 Mons, Belgium; alain.finet@umons.ac.be (A.F.); kevin.kristoforidis@umons.ac.be (K.K.)

**Keywords:** stock market simulation, financial emotions, virtual money, thematic analysis, behavioral finance

## Abstract

This article analyzes how novice investors emotionally experience a stock market simulation conducted with virtual money. Financial simulations are sometimes criticized because they do not reproduce the real financial consequences of investment decisions. However, the fifteen semi-structured interviews conducted after the simulation show that the absence of monetary loss does not eliminate the emotional intensity of the experience. Participants report stress, fear, frustration, disgust, disappointment, satisfaction, and pride. This article argues that virtual money shifts emotional responses toward other forms of issues. Virtual losses are often interpreted as signs of poor decision-making, a lack of competence, or personal failure. Conversely, virtual gains may produce feelings of satisfaction or validation of one’s choices. Ranking also plays a central role: it transforms the exercise into a competitive situation and gives social value to individual performance. The academic bonus constitutes another form of stake because it situates the simulation within a framework aimed at improving academic performance. Based on a thematic analysis of the interviews, this article identifies five dimensions: virtual money as partial protection against financial loss; ranking as a symbolic substitute for financial stakes, the academic bonus as a real consequence of performance, virtual loss as a perceived loss of competence and frustration as a driver of recovery attempts, withdrawal, or disengagement. These findings show that stock market simulations are useful tools for observing how novices assign emotional meaning to gains, losses, ranking and their own competence. This article contributes to the literature on emotions and experimental simulations by demonstrating that emotional intensity does not depend exclusively on exposure to financial loss. Even when virtual, money becomes emotionally significant when it is associated with stakes related to performance, social comparison, academic success, and decision-making identity.

## 1. Introduction

Stock market simulations play an important role in finance education and behavioral finance. They allow for the observation of investment decisions in a controlled environment, including buy and sell orders as well as time constraints that are more or less similar to financial markets. They provide a useful framework for analyzing how individuals, particularly novice investors, make decisions under uncertainty.

However, these simulations are subject to recurring criticism: participants do not risk their own money. The use of virtual capital may limit the scope of the results, as the emotions, trade-offs, and behaviors observed are not subject to the consequences of a real investment. This criticism ties into a broader debate in experimental economics regarding the role of monetary incentives. Camerer and Hogarth (1999) show that financial incentives can alter certain experimental behaviors, even though their effects vary depending on the nature of the tasks. Research on real monetary rewards highlights that decisions made with virtual money do not always produce the same behavioral and psychophysiological responses as decisions involving actual monetary gains or losses (Xu et al., 2018; Harrison, 2022).

This methodological distinction does not, however, imply that financial simulations lack emotional reality. Research on emotions in finance shows that investment decisions are rarely purely cognitive and are influenced by fear, hope, confidence, regret, frustration, or the desire to recoup a loss. Lo et al. (2005) emphasize that market fluctuations can elicit emotional reactions among traders. Similarly, research in behavioral finance highlights that the subjective experience of gain and loss plays a central role in decision-making.

The semi-structured interviews conducted following a four-hour stock market simulation confirm this paradox. The participants knew they were not risking their own financial assets. Some even described the simulation as a game or a situation in which they had “not much” to lose. Yet their accounts reveal intense emotions: anxiety, frustration, disgust, disappointment, satisfaction, or pride. Others associate a rise in their portfolio with pride in having made the right choices, while a decline results in disgust, panic, or a sense of failure.

This line of research is part of a broader program focused on stock market simulations, emotions, and financial decision-making behaviors among novice investors. Previous studies have shown that trading simulations can reveal strategic adjustments and competitive effects as the experiment progresses (Finet et al., 2026a). Other analyses have highlighted that trading styles are also associated with anticipated emotions and participant characteristics (Finet et al., 2026b). Finally, research on the instructional design of simulations underscores the importance of rankings and tournament-style incentives in participant engagement and behavioral responses (Finet et al., 2026c). This article builds on this research while shifting the analytical perspective and relying on a distinct four-hour simulation conducted with another cohort. Unlike previous studies, its primary objective is not to assess emotional dynamics or trading styles as such, but to understand how virtual capital can become a vehicle for strong emotions, judgments of competence, and social comparison.

The question is not whether a simulation perfectly replicates the emotional conditions of a real investment. It does not, precisely, because there is no financial loss. The question is why a situation involving no personal monetary loss can nonetheless evoke emotions. The hypothesis put forward in this article is that virtual money does not eliminate emotion but may redirect it toward other types of issues:

- A virtual loss can become a perceived loss of competence;
- Rankings can take the place of monetary stakes;
- An academic bonus can contribute to transforming the exercise into a situation with real-world consequences;
- Performance can affect one’s self-image as a competent or incompetent decision-maker.

The research question is as follows: why does a stock market simulation without actual financial loss nevertheless generate strong financial emotions among novice investors?

By answering this question, this article contributes to the literature on financial simulations and behavioral finance. The scientific value of simulations lies in their ability to reveal how participants emotionally invest in symbolic, competitive, and identity-related issues.

## 2. Literature Review

### 2.1. Financial Simulations: A Methodologically Controversial Teaching Tool

Financial simulations are frequently used in the teaching of finance and decision-making under uncertainty. They enable students to move from an abstract understanding of markets to a concrete decision-making experience. From this perspective, simulations are part of an experiential learning approach, where knowledge is constructed through action, observation of consequences, and reflection on experience.

Several studies highlight the value of simulations in finance. Wolmarans (2005) shows that financial management simulations can increase learner engagement and give students a greater sense of understanding concepts that are perceived as abstract. Farashahi and Tajeddin (2018) indicate that simulations are particularly effective for developing transferable skills, including problem-solving, interpersonal interactions, and self-awareness. Bakoush (2022) shows that simulation tools in finance can enhance student satisfaction, engagement, and their perception of more concrete learning. Recent studies confirm the growing relevance of simulation-based approaches in financial education. Online game-based financial education has been shown to improve students’ financial literacy in experimental settings (Cannistrà et al., 2024). Work on stock simulation trading courses also highlights the role of digital environments in supporting student motivation and learning outcomes (Chang et al., 2024). In addition, studies on digital financial education and learning-by-doing emphasize the role of interactive tools in supporting engagement and knowledge acquisition (Oberrauch & Kaiser, 2024).

In the field of behavioral finance, experimental and simulated settings also hold scientific value. Duxbury (2015a, 2015b) emphasizes that experiments make it possible to isolate certain behavioral mechanisms that are difficult to observe in real market data, including reactions to gains and losses or the influence of emotional states.

However, the use of virtual money constitutes an important methodological feature. In a simulation, participants can lose part of their fictitious portfolio without suffering any personal financial loss. This absence of risk calls for caution: the behavior observed in a simulation cannot be equated with those of investors risking their own capital. This distinction necessitates clarifying what simulations allow us to observe. Even though money is virtual, the experience can become subjectively meaningful depending on certain characteristics of the experimental design. The research challenge is to understand how fictitious money can become a vehicle for emotions, self-evaluations, and decision-making reactions.

### 2.2. Emotions in Financial Decision-Making: Affect, Loss, Frustration, and Confidence

Behavioral finance has demonstrated that financial decisions cannot be reduced to rational calculations of return and risk. Investors interpret gains, losses, and uncertainty through emotional expectations and reactions. Finucane et al. (2000) show that individuals often rely on rapid emotional impressions to assess the potential risks and benefits of a situation. Slovic and Peters (2006) also emphasize that risk, beyond cognitive analysis, is also experienced emotionally. The influence of emotional states on financial decision-making has also been highlighted by Kuhnen and Knutson (2011). Their work shows that positive emotional states can encourage risk-taking and boost confidence, while negative states can produce the opposite effects.

Financial emotions also manifest in the temporal dynamics of action. Fenton-O’Creevy et al. (2011) demonstrate that emotions and their regulation play a central role in decision-making in market situations. Even though participants in a simulation are not professionals, they are exposed to an emotionally active form of this dynamic and reinterpret their decisions based on their immediate consequences. Loss occupies a central place in this emotional experience. Shefrin and Statman (1985) show that investors may tend to sell winning securities too quickly and hold onto losing securities for too long. This phenomenon suggests that financial decisions are shaped by how individuals psychologically experience gains and losses.

Frustration is also a decisive emotion. It arises when the participant feels that a better decision could have been made. It thus reflects a discrepancy between what the participant thought they could do and what the simulation reveals about their performance. Conversely, satisfaction and confidence emerge when decisions appear to be validated by the market. A rise in portfolio value can be interpreted as proof of a good choice, even when that rise results from chance or an unanticipated market movement. Statman et al. (2006) show that overconfidence can be linked to trading activity, as investors sometimes overestimate their ability to correctly interpret the available information.

Thus, emotions reflect a confrontation with uncertainty, the constant evaluation of one’s choices, and the ambiguity of outcomes. Even when money is virtual, emotions are rooted in the experience of decision-making: having to choose without certainty, observing the consequences, comparing what happens with what might have happened, and then interpreting these results.

### 2.3. Symbolic Stakes, Ranking, and Decision-Maker Identity

To understand why a simulation without actual financial loss can generate strong emotions, the symbolic issues associated with the design must be considered. A stock market simulation can introduce evaluation, comparison, and recognition. Using rankings, academic bonuses, and the visibility of performance, these features can transform the exercise into a situation that is socially and identity-relevant.

Research on gamification sheds light on this point. Hamari et al. (2014) show that gamified systems can have positive effects on engagement, but that these effects depend on the context, the type of participants, and the mechanisms used. In a stock market simulation, the rankings function as a gamified mechanism: it facilitates comparison among participants and introduces a competitive dynamic. Hanus and Fox (2015), however, show that systems based on rankings can also produce ambivalent effects, particularly by accentuating social comparison and influencing motivation or the sense of competence.

Rankings thus play a central role in transforming virtual money into an emotional issue by giving performance a social value. This dynamic aligns with the work of Buunk and Gibbons (2007), who show that individuals frequently use others as reference points to assess their abilities.

Perceived competence is another essential element. Ryan and Deci (2000) identify the need for competence as one of the fundamental psychological needs that support motivation. In a stock market simulation, novice participants test their ability to understand, make decisions, and act appropriately in an uncertain environment. A loss may be experienced as a sign of incompetence or a misreading of the market. Conversely, a gain can reinforce the feeling of having made a good decision.

This aspect can be reinforced by the academic nature of the design. When the simulation is linked to an academic reward, the issues become partially real. Even though the capital is virtual, the bonus can affect the final exam grade. The experience thus lies at the intersection of several dimensions: a simulated financial dimension, an educational dimension, a competitive dimension linked to ranking, and an identity dimension linked to one’s self-image as a decision-maker.

Dweck’s (1999) work on implicit theories of ability also helps explain why the results of a simulation can be emotionally charged. When individuals interpret a performance as an indicator of personal ability, failure can undermine their self-image. In a stock market simulation, a bad result can be interpreted as evidence of an inability to understand the markets or make sound financial decisions.

This focus on competence contributes to the temporary construction of an identity as a decision-maker. Indeed, when participants link their gains or losses to their decision-making ability, they begin to define themselves through that outcome. They may perceive themselves as cautious, competitive, lucky, rational, unable to cope, or capable. Gains and losses thus become tools for self-interpretation.

This dynamic is reinforced by comparison with other participants. Tajfel and Turner (1979) show that situations involving comparison between individuals can help shape social identity and self-evaluation. In a simulation, participants position themselves relative to the performance of others. The emotional issues come from the meaning attributed to financial fluctuations in a context of visibility, comparison, and evaluation.

The stock market simulation can thus create a specific emotional reality. It brings into play forms of risk beyond financial risk: the risk of making a mistake, the risk of performing worse than others, the risk of not understanding, the risk of missing out on a bonus opportunity, and the risk of having one’s competence called into question. Virtual money thus becomes a vehicle for symbolic, competitive, academic, and identity-related stakes.

## 3. Methodology

### 3.1. Setting and Participants

The research was conducted at the University of Mons, at the Charleroi campus (Belgium) with students enrolled in the second year of the Bachelor’s program in Management Sciences. The participants had taken an introductory finance course prior to the simulation but had not yet taken the exam. For the purposes of this research, they are considered novice investors. This novice status is a key element of research design. It allows us to observe how individuals in the process of learning develop their decision-making frameworks when faced with uncertainty, price fluctuations, and comparisons with other participants. The objective is therefore to understand how students unfamiliar with stock market practices make sense of a simulated financial decision-making experience.

Twenty-five students participated in the stock market simulation. At the end of the simulation, all participants were invited to take part in a semi-structured interview. Fifteen students agreed to participate in the qualitative phase. The interview sample was homogeneous in terms of prior stock market experience. Fourteen of the fifteen interviewees reported no previous stock market experience, while one participant reported experience limited to a prior classroom-based simulation. This confirms the novice nature of the sample. Detailed participant characteristics are provided in Appendix G. Participation in the interviews was voluntary and depended on students’ agreement and availability after the simulation. The ten students who did not take part in the interviews did not provide reasons for declining. Their non-participation may have reflected practical constraints or limited availability after the simulation, but no reasons were formally collected.

To assess the possibility of self-selection bias, aggregate transaction indicators were compared between participants who were interviewed and those who were not, based on the available data. The comparison did not indicate substantial differences in exposure, portfolio variability, or return. Interviewees retained an average of 69.9% of the initial capital in cash, compared with 64.9% among non-interviewed participants. Portfolio value variability was also comparable, averaging EUR 14,739 among interviewees and EUR 17,835 among non-interviewees. Median returns were very close, at −0.41% among interviewees and −0.40% among non-interviewed participants.

Interviewed participants appeared somewhat more active, with an average of 19.0 transactions compared with 13.1 among non-interviewed participants. However, Mann–Whitney comparisons did not indicate statistically significant differences in transaction frequency (*p* = 0.215), portfolio value variability (*p* = 0.385), or return (*p* = 0.689). This suggests that the interview sample was not concentrated at one extreme of observed trading activity or return.

Our qualitative approach is consistent with methods focused on the meaning that participants attribute to their experiences, as described by Kvale and Brinkmann (2009). The descriptive characteristics of the fifteen semi-structured interviews are presented in Appendix F. The Appendix F provides details on the volume of qualitative data analyzed.

### 3.2. Stock Market Simulation

The stock market simulation took place on 4 December 2025, from 1:30 p.m. to 5:30 p.m. Its objective was to place participants in a financial decision-making situation structured by common rules, a time limit, and continuously accessible market information. Each student started with a virtual portfolio of 100,000 euros. Trades were executed on the ABC Bourse platform, in an environment configured specifically for the experiment.

Participants could buy and sell only securities included in the CAC 40 index. This choice limited the investment universe and ensured that decisions were comparable across participants. Short selling was not permitted, nor were limit orders. Participants were free to choose which securities to buy, the timing of transactions, the amounts invested, and the number of trades executed. A transaction fee of 0.2% was applied to each trade to introduce a constraint similar to real-market situation.

The system also included a leaderboard that was visible throughout the simulation. This introduced a competitive element into the experience and could influence how participants perceived their performance. A tournament-style incentive system was also incorporated into the simulation. The participant with the best performance received a 2-point bonus on the exam, the second, a 1.5-point bonus, and the third, a 1-point bonus. All participants also received 0.5 points for their participation.

The system thus combined three elements: virtual capital, a real academic incentive, and a continuously updated ranking. The instructions provided to participants before the start of the simulation, as well as a summary description of the trading environment offered by the ABC Bourse platform, are presented in Appendix D.

### 3.3. Market Context During the Simulation

The trading session on 4 December 2025 was characterized by a slightly positive trend in the CAC 40 throughout the day. The index closed up 0.42%, at 8122 points. However, during the simulation, the CAC 40 remained generally stable. The index rose from approximately 8120 points at the start of the simulation to approximately 8122 points at the end. Most of the session’s gains had occurred before the simulation began. The afternoon was more of a consolidation phase than a strongly directional market trend.

### 3.4. Qualitative Data Collection

The choice of semi-structured interviews was justified by the aim of accessing participants’ subjective experiences while maintaining a common structure across the interviews. This method makes it possible to address the same dimensions while giving participants the opportunity to freely develop their narratives, emotions, and interpretations.

This study relies on interviews rather than on matching narratives and transaction-level data because transaction records can indicate when an order was placed or how large a position was, but they do not show whether participants experienced the action as cautious, impulsive, frustrating, or recovery-oriented.

Several precautions were taken to limit recall bias. The interviews were conducted shortly after the simulation and structured around the chronological reconstruction of the experience. Questions were anchored in specific aspects of the simulation, such as portfolio changes, ranking movements, gains, losses, and the academic bonus, to encourage recall of situated moments rather than general impressions.

The interview guide covered several dimensions. Participants were first asked to describe their initial relationship with the stock market and risky financial decisions. They were then asked about their state of mind before the simulation began, the emotions they felt during the experience, how they reacted to gains and losses, and the criteria they used to decide whether to buy, sell, or hold a position. The guide also addressed the role of rankings, comparisons with other participants, and the academic bonus. The interview guide used to structure the discussions is presented in Appendix E.

### 3.5. Choice of Analytical Method

A reflexive thematic analysis was conducted on the fifteen semi-structured interviews to examine how novice investors emotionally interpret gains and losses. This approach allows for the identification of recurring patterns of meaning in the narratives while preserving the complexity and contextualization of individual experiences. Thematic analysis is a flexible method for identifying, organizing, and interpreting recurring themes in a qualitative corpus (Braun & Clarke, 2006, 2021).

The analysis combines two complementary approaches. The first is inductive: codes are initially constructed based on the participants’ statements, without imposing a rigid theoretical framework. The second is abductive: the empirical data are then interpreted considering the article’s research question. Abduction allows the data to be linked to the conceptual framework without reducing the analysis to a straightforward application of preexisting categories. This approach is consistent with the proposals of Timmermans and Tavory (2012), who argue that abductive analysis aims to produce a theoretical interpretation based on empirical elements that require explanation.

### 3.6. Operational Analysis Procedure

The analysis was conducted in eight steps.

The first stage involved preparing the corpus. The fifteen interviews were transcribed to facilitate their processing. This stage made it possible to identify passages related to the research focus: attitudes toward virtual money, reported emotions, reactions to wins and losses, the role of rankings, academic bonuses, and interpretations of performance.

The second stage involved familiarization with the data. Each transcript was read several times to identify the overall logic of the narrative, the key moments of the experience, and the tensions expressed by the participants. This familiarization phase allows the researcher to develop a detailed understanding of the material before generating the initial codes (Braun & Clarke, 2006). Analytical notes were taken throughout the readings to record initial impressions and potential lines of interpretation.

The third step consisted of descriptive coding. Interview segments were coded using categories grounded in the participants’ own wording. The descriptive codes aimed to identify named emotions, stated reactions, and elements explicitly mentioned in the narratives. This initial coding included emotions experienced, reactions to gains and losses, references to rankings, mentions of the academic bonus, judgments on the quality of decisions and changes in strategy.

The fourth step consisted of interpretive coding. The descriptive codes were revised to identify their meaning within the narrative. For example, a stated emotion was considered an indicator of decision-making uncertainty, a loss of control, an unfavorable comparison, a sense of error, or a skill. This step facilitated a shift from descriptive to analytical reading. It corresponds to an approach to interpreting meaning, similar to what Miles et al. (2014) describe as a process of reducing and relating qualitative data.

The fifth step involved grouping the codes into temporary themes. Descriptive and interpretive codes were compared across interviews to identify recurring patterns. The themes were defined based on their relevance to the research question. In other words, a theme was selected when it contributed to understanding how virtual money could become emotionally significant despite the absence of any actual financial loss. This phase involved repeated comparisons between interview verbatims, codes, provisional themes, and the article’s research question.

The sixth step consisted of constructing a cross-case matrix. For each participant, several dimensions were identified: initial relationship to the stock market, relationship to virtual money, dominant emotion, reaction to gains and losses, importance placed on rankings, role of the academic bonus, perception of personal competence, and interpretation of the experience. The purpose of this matrix is to strengthen the coherence of the analysis by enabling a comparison among the fifteen cases and verifying the robustness of the interpretations (Miles et al., 2014).

The seventh step involved finalizing the themes. The temporary themes were reviewed based on three criteria: their internal consistency, their distinction from other themes, and their ability to address the research question. This step helped clarify the analytical contribution of each theme. It is part of a commitment to qualitative rigor, where the analysis must document the interpretive process followed by the researcher. Nowell et al. (2017) emphasize the importance of an analytical trail to strengthen the credibility and transparency of the analysis. To make this transition from codes to final themes more transparent, a summary of the thematic grouping process is presented in Appendix C.

Finally, the eighth step involved selecting verbatim excerpts for the presentation of the results. They served to illustrate the interpretive mechanisms identified. The verbatim excerpts were used as empirical support for the analysis, without reducing the results to a juxtaposition of quotations. The rationale for selecting the verbatims is illustrated in Appendix B, which presents, for each theme, a few representative verbatim quotes along with their analytical function.

To illustrate how the analytical procedure was applied to the empirical material, Appendix A presents a detailed example based on one individual interview.

### 3.7. Coding Credibility and Researcher Positionality

The reflexive thematic analysis involved the three authors. A preliminary subset of interview extracts was first coded independently by the researchers. The purpose of this stage was to compare interpretations, clarify the meaning of the codes, and identify potentially ambiguous passages. Divergences mainly concerned the interpretation of emotions that could carry more than one meaning and the distinction between descriptive and interpretive codes.

Differences in interpretation were discussed collectively by returning to the full interview context, the participant’s wording, and the surrounding narrative sequence. When necessary, the codebook was refined and coding rules were clarified before being applied to the full corpus. Remaining ambiguous cases were resolved through discussion and consensus. Given the interpretive and non-exclusive nature of the thematic categories, no formal inter-coder reliability coefficient was calculated.

Although this study was conducted in an academic setting, the interviews were conducted by a researcher who had no teaching relationship with the participants and was not involved in their course assessment. Participation in the interviews was voluntary and was separated from the academic bonus and course assessment. These precautions were intended to reduce the influence of power relations on participants’ statements and to encourage them to describe their experience freely.

## 4. Results

The thematic analysis of the fifteen interviews highlights that participants are aware that the money used in the simulation is not real. However, virtual money does not eliminate the frustration, disappointment, satisfaction, or pressure associated with rankings or academic bonuses. Emotions seem to be redirected toward other issues: position in the rankings, validation of the choices made, and self-image.

The five themes presented below result from a cross-case comparison. Some interviews primarily contribute to one theme, while others contribute to several dimensions. Table 1 summarizes the thematic framework.

A review of this matrix shows that the most frequent themes are those related to frustration, regret, and the desire to recover (T5), present in thirteen interviews, and those related to virtual loss as a perceived loss of competence (T4), present in twelve interviews. T1 appears in nine interviews, while T2 and T3 are each present in seven interviews. Reactions to loss and the assessment of competence thus emerge as the most cross-cutting dimensions.

### 4.1. Virtual Money as Partial Emotional Protection, but Not as Emotional Neutrality (T1)

The first theme highlights the ambivalent role of virtual money. Several participants emphasize that the simulation does not expose them to personal financial loss. However, this absence of financial risk does not transform the simulation into an emotionally neutral experience.

P14 explains that at first she felt stressed, but then she kept in mind that she had already earned half a point and that she was not “really gambling with money.” This fact reduces the pressure. Yet her account evokes positive stress, a sense of control when she sees the consequences of her choices, followed by a reaction to the rankings, which she experienced as both stressful and reassuring.

P13 explains that knowing it is a “fake portfolio” helps her maintain a trial-and-error mindset: “It’s not real money anyway.” She adds that if real money had been at stake, she would have been “even more careful.” In other words, the “fake” portfolio changes the way participants allow themselves to experiment, learn, and make decisions.

Virtual money appears to act as a partial protective factor, but it does not eliminate the emotions associated with uncertainty, performance, or comparison. The real financial dimensions are absent, but other stakes take their place.

### 4.2. Rankings as a Substitute for Financial Stakes (T2)

The second theme shows that ranking plays a central role in transforming the simulation into an emotional experience. Ranking allows students to assess their standing relative to one another and may introduce a competitive dimension that may partly substitute for real financial consequences.

P10 explains that she initially entered the simulation telling herself she had “nothing to lose” and that it was not “even about money.” But when the rankings are displayed, her relative position changes her level of commitment: she notices that she is initially eleventh, then moves up, which motivates her “just a little more.” The rankings may therefore turn virtual performance into a source of motivation.

For P9, the rankings are directly linked to comparison and perceived competence. When he sees that the other participants have a lower portfolio value, he feels more confident. He explains that the ability to compare himself to others motivates him to continue learning how the stock market works, because he feels he has figured out “how it all works.” The ranking is interpreted by this participant as an indicator of competence.

P12 describes the role of the ranking as a source of satisfaction and challenge. Being well-positioned brings satisfaction, and the ranking generates “positive stress,” experienced as a challenge. Comparing oneself to others can sustain engagement, boost confidence, and reinforce the feeling that a strategy is effective.

In this simulation context, rankings can act as a symbolic substitute for real money. They enable social comparison and may turn the simulation into a competitive situation. Even though the capital is virtual, one’s position can become emotionally significant.

The role of rankings must also be understood within the specific context of the audience. The simulation takes place in a small group composed of students who know one another and have a sense of each other’s academic performance. In this type of group, comparisons are part of everyday social interaction, shaped by personal relationships, academic reputations, implicit rankings, and expectations regarding everyone’s abilities.

The stock market simulation, however, introduces a different kind of ranking. Academic performance does not guarantee success in the experiment. A student usually perceived as high performing may rank poorly, while another, less commonly identified as a “good student,” may achieve a higher position. The simulation’s ranking may temporarily shift the class’s usual hierarchies. It creates a specific arena for comparison, based on the ability to make decisions in an uncertain environment.

From this perspective, participants observe their position within a group where comparison holds social significance. The value of the virtual portfolio may become an indicator of success and a means of temporarily repositioning oneself within the class.

### 4.3. The Academic Bonus as a Real Consequence of the Simulation (T3)

The bonus may transform the exercise into a partially real-world situation, as it can affect the exam grade. For P15, the bonus point is explicitly associated with enthusiasm and relief. She explains that the prospect of earning a bonus point makes the simulation “more enjoyable” and provides “relief,” particularly because the exam is perceived as difficult. She clarifies that she does not necessarily aim to be in the top 3, but that the guaranteed half-point reduces some of the pressure.

For P13, the guaranteed half-point also helps reduce some of the initial stress. She viewed the experience as a game and knew that all participants already had a half-point. This minimal reward transforms the experience into a positive situation: even without finishing among the top scorers, participating “will only bring [her] positive outcomes”.

However, the bonus can have the opposite effect when combined with academic pressure. For P9, the two points at stake are presented as very important, particularly because he had previously failed the exam. He distinguishes between positive stress (linked to the feeling of being able to handle the simulation) and negative stress (linked to the possibility of not earning those two points). The bonus becomes a real academic issue.

The academic bonus may therefore alter the nature of what is at stake. Participants may wonder whether they will earn points or take advantage of an academic opportunity.

### 4.4. Virtual Loss as a Perceived Loss of Competence (T4)

Virtual losses are often experienced as signs of poor decision-making, a lack of understanding, or insufficient competence.

For P3, the portfolio turning negative is described as “a big shock.” When asked how he feels when the portfolio drops, he replies that he feels “lost.” The virtual loss causes disorientation and calls into question his ability to make decisions in an environment he is unfamiliar with.

For P6, the portfolio’s value serves as an indicator validating her choices. When stock prices rise, she feels happy and “pretty proud.” When the value falls, she says she feels “disgusted” and that it makes her panic. A gain validates the decision, and a loss invalidates it. Virtual performance becomes an immediate measure of the perceived quality of the decision.

P12 defines a good decision as “making a profit” and a bad decision as “making a loss.” This equivalence shows that participants tend to judge their decisions based on the outcome, even when that outcome also depends on volatility or chance.

P11 also attributes the decline in his portfolio to a “lack of information.” He interprets a decision as having “cost him the win” and associates stress with stock price volatility and not knowing what to do. In this narrative, virtual loss is interpreted as a sign of a lack of understanding.

This theme suggests that virtual money can involve perceived skill. The loss may affect one’s self-image as a decision-maker. Participants do not simply interpret the loss as “I lost some fictional money,” but rather as “I made a bad choice,” “I didn’t understand,” “I wasn’t paying enough attention,” or “I didn’t know how to react”.

### 4.5. Frustration, Regret, and the Desire to Recoup Losses (T5)

The interviews show that a decline in the portfolio’s value can result in reactions similar to real-life financial situations: regret at not having sold, a desire to recoup losses, a desire to make up for it, or frustration at having made the wrong choice.

When discussing the decline, P1 says he feels “disgusted” and is looking for “a way to make up for it.” He adds that he has a hard time accepting the loss and is still looking for a way to offset it. Later, he explains that after losing money, he increased the amount invested in another stock to break even: he went from investing 10,000 euros to 25,000 euros, acknowledging that by then he had “lost all sense of money.”

P3 describes another form of catch-up behavior. After incurring a loss, he says he feels “a little frustrated” and wants to “absolutely make it back.” In his case, virtual loss generates a desire to recover and makes it difficult for him to remain calm as his emotional state deteriorates.

P6 expresses this logic of recovery and gradually links it to discouragement. Faced with a loss, she says she feels “disgusted” while telling herself that she can “make it up later.” However, toward the end of the simulation, she feels rather discouraged because she no longer believes she can recoup the loss. The logic of recovery can turn into withdrawal when recovery seems impossible.

P8 describes a trajectory even more marked by a loss of confidence. As the value of her portfolio declines, she speaks of sadness and a “loss of confidence.” Toward the end, she says she feels “sickened” because the value of her portfolio “keeps going down, it just keeps going down.” This decline affects her morale and leads her to withdraw from the experiment.

This theme shows that virtual loss produces several types of reactions. Some participants try to recover their losses, others regret a sale or a purchase, and others become discouraged. In all cases, virtual loss is not emotionally neutral and may become an event to be corrected or avoided.

## 5. Discussion

This discussion proposes interpreting these results based on five theoretical frameworks.

### 5.1. From the Absence of Monetary Loss to Self-Testing

The first contribution of this article is to suggest that the absence of financial loss does not mean the absence of loss. In the interviews, participants know that the money is virtual. Yet their accounts link fluctuations in the value of the portfolio to feelings of pride, disgust, frustration, panic, disappointment, or a loss of confidence. Virtual loss may become an interpretive event. It may be interpreted as a sign of a bad choice, a lack of competence, or an inability to properly understand the market.

This finding can be linked to the theory of self-discrepancy. Higgins (1987) shows that discrepancies between how individuals perceive themselves and personal standards can generate negative emotions. In the context of the simulation, the virtual loss can be interpreted as a discrepancy between the decision-maker the participant hoped to be and the decision-maker he feels he was. This interpretation supports the idea that virtual loss represents a perceived loss of competence. When some participants say they are proud of their choices when the portfolio’s value rises, or feel lost when it falls, they interpret the outcome as an indicator of competence. This mechanism is consistent with the work of Heath and Tversky (1991) on competence in situations of uncertainty: individuals are more inclined to engage in uncertain choices when they feel competent. The novice investors studied are placed in a situation where their competence is fragile. This helps explain why virtual losses may become emotionally sensitive, as they appear to confirm a lack of control or insufficient competence.

### 5.2. The Virtual Portfolio as an Object of Psychological Attachment

The second contribution of this article concerns the status of the virtual portfolio. Participants do not own the money invested, but their narratives show that they can nevertheless become attached to it. They monitor fluctuations, wait for the value to rise, derive satisfaction from gains, worry about declines, and sometimes seek to recoup what has been lost. The narratives suggest the emergence of a form of psychological ownership.

Pierce et al. (2001) define psychological ownership as a state in which an individual feels that an object or entity is “theirs,” even in the absence of formal ownership. In the simulation, the portfolio is assigned to the participant, evolves because of their decisions, and becomes a visible manifestation of their choices. This configuration may encourage a sense of subjective ownership through which the portfolio is experienced as “my portfolio.” This sense of ownership may help explain why virtual gains and losses can evoke strong emotions. A decline affects an object with which the participant has temporarily identified. Similarly, an increase can be experienced as validation of “my choices” and “my strategy”.

### 5.3. Rankings as Symbolic Capital and a Space for Self-Presentation

The results show that the ranking can operate as a substitute for monetary dimensions and may turn the simulation into a situation of comparison. This dynamic can be interpreted through the concept of symbolic capital. According to Bourdieu (1986), certain resources acquire value when they are socially recognized as legitimate. In the simulation, the ranking may function as a form of provisional symbolic capital. It produces relative recognition, and the virtual result becomes socially legible.

Ranking can also be understood as a form of self-presentation. Goffman (1959) shows that social interactions involve the management of one’s self-image in the presence of others. Simulation exposes participants to comparative evaluation, and ranking may enable a social interpretation. In the simulation, emotion is also shaped by the fact that participants know they are being observed and compared by others.

### 5.4. The Academic Bonus as a Concrete Goal and a Transformation of the Simulation’s Status

Even though the money is virtual, the bonus introduces a real consequence within the academic setting. This dimension can be linked to goal-setting theory. Locke and Latham (2002) show that specific goals can direct effort, attention, and persistence. The academic bonus may function as a goal that gives concrete value to performance. This goal can produce relief when a minimum is guaranteed but also stress when the extra points are perceived as important.

Regulatory focus theory also helps explain the coexistence of two different experiences of the bonus. Higgins (1997) distinguishes between a promotion focus, oriented toward achievement and gain, and a prevention focus, oriented toward avoiding failure. The bonus can activate both responses. For some participants, it makes the simulation more enjoyable because it offers an opportunity for academic recognition. For others, it increases the pressure because it becomes a resource they cannot afford to miss out on. The same mechanism can generate enthusiasm, relief, or anxiety depending on how the participant interprets the academic consequences of the simulation.

### 5.5. Loss Recovery and the Search for Symbolic Balance

The fifth contribution of this article concerns reactions to virtual loss. Several participants describe a desire to make up for a loss or correct a bad decision. Virtual loss can generate compensatory behaviors similar to decisions involving real money.

The work of Thaler and Johnson (1990) helps clarify this mechanism. Their analysis of the search of equilibrium shows that prior losses can encourage risk taking aimed at returning to the starting point. In the interviews, the use of language related to “making up for losses” suggests that the loss is experienced as a deviation that must be compensated for.

This dynamic can also be linked to the illusion of control. Langer (1975) shows that individuals may overestimate their ability to influence uncertain outcomes, particularly when the situation involves elements of competition or personal involvement. In the simulation, participants choose the securities, the timing of transactions, and the amounts invested. Those features may reinforce the impression that a loss can be recouped through a subsequent decision. The market context further qualifies this interpretation. Although the CAC 40 remained broadly stable during the four-hour session, participants did not describe the experience as emotionally neutral. Their narratives suggest that, in the absence of a clear directional trend, attention shifted toward small portfolio movements, isolated price changes, and the immediate consequences of individual decisions. This context may have strengthened the tendency to consider small gains and losses as consequences of one’s own choices, thereby reinforcing hesitation, frustration, or recovery-oriented reactions.

## 6. Conclusions

The aim of this article was to understand why a stock market simulation without actual financial loss can elicit emotions in novice investors. To answer this question, we analyzed fifteen semi-structured interviews conducted following a four-hour stock market simulation. The analysis was conducted using a thematic–reflexive approach, combining inductive coding of the narratives with abductive interpretation focused on the symbolic, competitive, academic, and identity-related issues associated with virtual money.

The approach was based on the participants’ expressed experiences. The interviews were first reviewed to identify reported emotions, reactions to gains and losses, attitudes toward virtual money, the role of rankings, and the significance of the academic bonus. The descriptive codes were then refined into interpretive codes to understand what the emotions signified within the narratives: feelings of competence or incompetence, comparisons with others, frustration over losses, a desire to recover, or disappointment with performance. Finally, a cross-case comparison identified five overarching themes.

The answer to the research question is as follows: a stock market simulation using virtual money elicits emotions because the absence of real financial loss does not mean that the experimentation has no meaningful consequences. Virtual money does not seem to make the experience emotionally neutral. Virtual gains and losses may be interpreted as signs of successful or failed decision-making. A rise in portfolio value can elicit satisfaction, pride, or a sense of mastery, while a decline can produce disgust, frustration, and a loss of confidence.

The results also show that rankings and academic bonuses are relevant components in stock market simulation when it comes to eliciting emotions. Rankings may introduce a form of social comparison that gives emotional value to the portfolio. The academic bonus shifts the focus to academic success and the opportunity to earn points. These two elements reinforce participants’ engagement and help make the simulation an emotionally meaningful experience.

These findings have practical implications for financial education. While virtual money can encourage engagement, experimentation, and active learning, it may also reveal frustration, impulsive recovery attempts, or withdrawal when gains and losses are interpreted as signs of competence or failure. From a pedagogical perspective, simulations may therefore help students identify emotional and behavioral patterns that could, in other contexts, contribute to maladaptive financial decisions. Educators should include structured debriefing phases focused on decisions, emotions, and performance interpretation. Rankings can support engagement, but they should be carefully framed so that competition does not become the sole purpose of the exercise. Similarly, academic bonuses should remain limited in weight to motivate participation without allowing short-term performance to overshadow the learning objective.

## 7. Limitations and Directions for Further Research

This research has several limitations that must be considered when interpreting the results.

The first limitation relates to the specific nature of the design. The simulation studied combined several components: a virtual portfolio, a time limit of four hours, an investment universe restricted to the CAC 40, a visible ranking, and an academic bonus. The results suggest that the ranking and the bonus play an important role in the emergence of emotions. However, this combination makes it difficult to isolate the specific effect of each component. The emotions observed cannot therefore be attributed solely to virtual money, the ranking, or the bonus, but rather to the interplay of these elements within the same situation.

A second limitation concerns the participants’ profile and the institutional context of the study. The research was conducted with novice investors studying at a single university and participating in one educational stock market simulation. The aim is not to generalize statistically from this sample, but to offer transferable insights into how novice investors may experience comparable educational simulations. By providing detailed information on the participants, the simulation design, the interview procedure, and the analytical process, this study allows readers to assess whether the mechanisms identified may be relevant to comparable educational simulations involving novice investors. The design could also be applied to other investor profiles, such as experienced retail investors, market professionals, or students from other disciplines. However, these groups might attribute different meanings to virtual money and might react differently to virtual gains, losses, rankings, and academic or non-academic incentives.

A third limitation concerns the duration of the simulation and the market conditions during the session. The experiment lasted four hours and took place in a relatively stable market context. This format may underrepresent the intensity of reactions associated with sharp market declines or strong upward movements, such as panic or euphoria. The emotional patterns identified should therefore be understood in relation to a short educational simulation with limited market fluctuations.

A fourth limitation concerns the data collection method. The interviews were conducted after the simulation. This approach does not allow for the measurement of emotions in real time during the simulation. Reported emotions may be influenced by memory or by how participants wish to present their experience in retrospect.

These limitations open several avenues for further research. One approach would be to compare different configurations of the stock market simulation. It would be useful to observe whether emotions vary depending on whether the simulation includes a visible ranking, an academic bonus, a real monetary reward, or simply a learning-focused format without evaluation. Such a comparison would provide a better understanding of the specific role that each component plays in frustration, pride, or the desire to recover.

A second approach would be to compare the reactions of novice investors with more experienced investors. It would be interesting to examine whether more experienced participants are better able to dissociate performance from their perceived competence, or whether they are affected by the ranking, the virtual loss, and the desire to recover.

A third avenue of research would involve combining interviews with data collected during the simulation. Real-time measures, such as repeated self-assessments, behavioral observations, or physiological indicators, would provide a better understanding of the temporal dynamics of emotions. This would help distinguish between emotions experienced at the moment of decision-making and those reconstructed during the interview.

A fourth approach would focus on the link between reported emotions and trading behaviors. Further research could cross-reference interview narratives with transaction data: number of orders, position sizes, timing of buys and sales, reactions following a loss, and risk-taking after a gain or a decline in portfolio value.

Finally, a fifth line of research could examine the educational dimension of the design. While rankings and academic bonuses may help elicit emotional responses, they can also influence how participants learn, take risks, or interpret their mistakes. Future research could examine the educational effects of these components: do they encourage engagement and help participants retain the experience, or do they increase pressure, social comparison, and certain catch-up behaviors? This question is important for designing stock market simulations capable of facilitating experiential learning, while ensuring that competition or rewards do not take precedence over critical thinking.

## Figures and Tables

**Table 1:** Condensed Cross-Case Matrix of Contributions to Themes.

Participant	Main Contribution to the Analysis	Topics Addressed
P1	Frustration and recovery after a virtual loss; loss experienced as a disappointing performance	T1, T4, T5
P2	Regret at not having sold sooner; correction under stress	T4, T5
P3	The shift into the red was experienced as a shock and a loss of bearings	T1, T4, T5
P4	Academic bonus, initial anxiety, and frustration mitigated by play money	T1, T3, T4, T5
P5	Virtual loss as self-disappointment and gradual abandonment	T4, T5
P6	Increase associated with pride in one’s choices; decrease associated with disgust, panic, and catching up	T1, T4, T5
P7	Stress, ranking, and regret despite the absence of financial loss	T1, T2, T3, T4, T5
P8	Loss of confidence, disillusionment, and withdrawal in the face of a declining portfolio	T4, T5
P9	Bonuses and rankings as major academic and competitive challenges	T2, T3, T4
P10	Shift from a “no-stakes” mindset to competitive engagement tied to rankings	T1, T2, T5
P11	Loss interpreted as the result of a lack of information and a poor choice	T4, T5
P12	Bonuses, rankings, and performance as criteria for success	T2, T3, T4, T5
P13	Simulation experienced as a controlled game, but rankings as a real-world comparison	T1, T2, T3
P14	Virtual money and the guaranteed half-point as protective factors; visible rankings and consequences as emotional engagement	T1, T2, T3, T4, T5
P15	Academic bonus, relief, and progressive learning through ranking	T1, T2, T3, T5

Legend: T1 = virtual money as partial protection; T2 = ranking as a substitute for monetary stakes; T3 = academic bonus as a real consequence; T4 = virtual loss as a perceived loss of competence; T5 = frustration, regret, and desire to recover.

## Data Availability

The original contributions presented in this study are included in the article. Further inquiries can be directed to the corresponding author.

## Appendix A

### An Example of the Application of the Analysis Procedure: The Case of P1

This appendix illustrates, based on an individual interview, how the thematic analysis procedure was applied to the empirical data. It does not include all the collective steps described in the Methodology Section (in particular, the cross-case comparison and the final consolidation of themes) which were carried out across all fifteen interviews. It shows the progression from an interview to the initial levels of analysis: identifying relevant passages, reconstructing the emotional trajectory, descriptive coding, interpretive coding, formulating provisional individual themes, and producing a summary sheet to feed into the cross-case matrix.

1. Identifying Relevant Passages

P1’s interview was analyzed based on the dimensions selected for the entire corpus: initial attitude toward the scholarship, emotional state before and during the simulation, reactions to gains and losses, attitude toward virtual money, the influence of emotions on decisions, and interpretation of performance.

The participant explains that he had already heard of the stock market but had “never really been interested” in it. He clarifies that he told himself the stock market “wasn’t necessarily for people like him” and that he “might not have the necessary skills and knowledge to invest.” He therefore approaches the simulation with curiosity and doubts about his financial competence.

The virtual nature of the money initially serves as a safeguard: the participant describes himself as “excited” and states that “winning or losing isn’t a big deal.” However, the rest of the story shows that this initial detachment does not eliminate emotional involvement. He thought he would “make a lot of profit,” but the experience gradually turns into disappointment, frustration, and a desire to bounce back.

2. Emotional Trajectory

The participant’s emotional trajectory is clearly downward. He sums up his experience as follows: “At first, I was excited. In the middle, I was demoralized. And at the end, I was a little disgusted, a little disappointed with my performance”.

The rise in the portfolio’s value is associated with satisfaction and excitement: the participant says he feels “happy,” “as if it would never stop going up.” He also explains that he was watching the fluctuations “down to the last cent,” which indicates that the virtual portfolio becomes the focus of intense attention.

Conversely, a decline in the portfolio’s value is associated with disgust, frustration, and the need for recovery: “I was disgusted, and I was looking for a way to make up for it. I don’t really accept the loss: I’m always looking for a way to try to make up for it.” The virtual loss is therefore not experienced as a virtual fluctuation, but as a situation that needs to be corrected.

3. Descriptive and Interpretive Coding

**Table A1:** Illustrative verbatim quotations, descriptive codes, and interpretive codes resulting from the thematic analysis of participants’ experiences during the stock market simulation.

Verbatim	Descriptive Code	Interpretive Code
“Maybe I didn’t have the necessary skills and knowledge to invest.”	Doubts about financial competence	The stock market is perceived as a demanding world, reserved for those with knowledge and resources.
“Winning or losing isn’t a big deal.”	Initial minimization of the consequences	Virtual money initially reduces fear of losing one’s wealth.
“I thought I was going to make a lot of money.”	Anticipation of success	The participant enters the simulation with positive expectations that are not fulfilled.
“Frustration. Mostly frustration.”	Overwhelming frustration	The gap between initial expectations and the actual outcome becomes the emotional focus.
“I was watching every penny.”	Intense monitoring of the portfolio	The virtual portfolio becomes a focus of attention and emotional investment.
“Happy. As if it would never stop going up.”	Satisfaction with the rise	The rise is experienced as validation of the choices made.
“Disappointed with my performance.”	Disappointment with performance	The result is interpreted as a personal performance, not as a random fluctuation.
“I don’t really accept the loss.”	Rejection of the loss	The decline is emotionally rejected and prompts a reaction.
“I was looking for a way to make up for it.”	Desire to make up for the loss	The loss generates a desire to recover what has been lost.
“I had lost all sense of money.”	Increased commitment	The virtual nature of capital can encourage increased risk-taking after a loss.
“These were decisions I made because I was frustrated and wanted to get back on my feet.”	Decisions made out of frustration	The participant states that frustration influenced some of his decisions to recoup his losses.

4. Interpretation of the Case

The analysis shows that the virtual nature of the simulation primarily serves as a protective factor: the participant knows he is not risking his own assets and enters the simulation with curiosity. However, this protection does not eliminate emotional involvement. As soon as the portfolio’s value changes, it becomes a source of anticipation, satisfaction, frustration, and self-assessment.

The case highlights the shift from a mindset of discovery to one of recovery. If the portfolio’s value rises, the participant seeks to prolong the success. When the value falls, the objective changes: it is no longer just about winning, but about “making up for losses.” Virtual loss becomes a negative outcome to be corrected.

This case also shows that the loss is interpreted as a disappointing performance. The participant does not merely state that he has lost virtual money and he says he is “disappointed with [his] performance” and reinterpret certain decisions as “bad buys.” The value of the virtual portfolio functions as an indicator of perceived competence.

5. Preliminary Individual Themes

**Table A2:** Preliminary individual themes identified from the thematic analysis, with representative verbatim quotations and their corresponding interpretations.

Preliminary Individual Theme	Supporting Verbatim Quotes	Interpretation
Entry into a safe environment	“Excited”; “Winning or losing isn’t a big deal”	The simulation is initially experienced as a journey of discovery, protected by the absence of any real financial loss.
Anticipation of success	“I thought I was going to make a lot of profit”	The participant enters the simulation with positive expectations that will later be dashed.
Virtual gains as emotional stimulation	“Happy”; “as if it would never stop going up”	The rise in the portfolio’s value brings satisfaction, excitement, and a desire to continue.
Paper losses as a result of disappointing performance	“Demoralized”; “disgusted”; “disappointed in my performance”	A decline in the portfolio’s value is interpreted as a personal failure or a mistake in decision-making.
Emotional recovery	“I was looking for a way to make up for my losses”; “Now I have to make up for my losses”	The loss triggers a desire to make up for it.
Decisions influenced by frustration	“These were decisions I made because I was frustrated.”	The participant acknowledges that emotion helped guide certain decisions.

6. Participant Summary Sheet

**Dimension**	**Summary**
Initial attitude toward the stock market	Little familiarity with the stock market; curiosity, but doubts about his skills.
Initial Mindset	Excitement, curiosity, and positive anticipation.
Attitude toward virtual money	Virtual money initially protects against financial anxiety but does not eliminate emotional involvement.
Dominant emotion	Frustration.
Emotional trajectory	Initial excitement, demoralization in the middle, disgust and disappointment at the end.
Reaction to gains	Satisfaction, excitement, and a desire to see the value continue to rise.
Reaction to losses	Disgust, denial of the loss, and a desire to recoup the loss.
Emotion–decision link	Emotions, particularly frustration, are recognized to influence decisions.
Perceived competence	A decline in the value of the portfolio is interpreted as disappointing performance and as a sign of poor choices.
Summary interpretation	For this participant, the virtual loss becomes a disappointing performance, and then a matter of recovering what has been lost. The participant is not seeking to earn more; above all, he is seeking to erase the loss and restore an image of himself as a capable decision-maker.

## Appendix B

### Illustrative Verbatim Quotes by Theme

**Table A3:** The final themes identified through the thematic analysis, with illustrative verbatim quotations and their analytical functions.

Theme	Illustrative Verbatim Quotes	Analytical Function
T1. Virtual money as partial protection	“Winning or losing isn’t a big deal”; “I wasn’t really gambling”; “Anyway, it’s not real money.”	Shows that the virtual nature of the money allows for an initial sense of detachment, without preventing emotional involvement.
T2. Ranking as a substitute for monetary stakes	“When I saw that I’d moved up, it motivated me even more”; “It creates stress, but positive stress—more like a challenge.”	Shows that the ranking makes performance visible and emotionally engaging.
T3. Academic bonus as a real consequence	“The bonus point […] makes it a little more enjoyable”; “Two points is huge”; “We all had half a point.”	Shows that the real issues are not financial, but academic.
T4. Virtual loss as a perceived loss of competence	“I was pretty proud of my choices when the value was going up”; “I was lost—I didn’t know how to react”; “A bad decision is one that results in a loss.”	Shows that portfolio fluctuations are interpreted as an indicator of competence or error.
T5. Frustration, regret, and the desire to recover	“I was looking for a way to make up for it”; “I absolutely wanted to recover”; “I can make it up later.”	Shows that virtual loss generates reactions of recovery, regret, or withdrawal.

## Appendix C

### Transition from Preliminary Themes to Final Themes

**Table A4:** The development of the final themes from the preliminary coding, showing the progression from preliminary themes to analytical groupings and the final themes retained.

Preliminary Themes Derived from Coding	Analytical Grouping	Final Theme Selected
Virtual money; no real loss; simulation as a game; reduced financial anxiety	Virtual capital reduces certain risks but does not neutralize emotional engagement	T1. Virtual money as partial protection, but not as emotional neutrality
Ranking; comparison with others; relative motivation; stress related to rank; position within the group	Virtual performance becomes socially visible and competitive	T2. Ranking as a substitute for monetary stakes
Guaranteed half-point; bonus points; academic opportunity; exam pressure	The simulation produces a real-world consequence beyond the financial dimension	T3. The academic bonus as a real consequence of the simulation
Poor choices; lack of information; loss of confidence; self-disappointment; underperformance	Virtual loss is interpreted as a sign of competence or incompetence	T4. Virtual loss as a perceived loss of competence
Frustration; regret; desire to try again; recovery; withdrawal; giving up	Loss generates reactions of recovery or disengagement	T5. Frustration, regret, and desire to try again

## Appendix D

### Instructions for Participants and a Summary Description of the ABC Bourse Platform

The following instructions were provided to participants before the simulation began and were verbally reiterated to ensure a clear understanding of the general rules.

1. Objective of the simulation

Participants took part in a four-hour trading simulation on the ABC Bourse platform. Each participant managed an individual virtual portfolio with an initial balance of 100,000 euros. The objective was to maximize the final value of the portfolio within the incentive system described in the article.

2. Trading Environment

The simulation was conducted in an individual trading environment. Participants could buy and sell stocks included in the CAC 40 index. Orders were executed at the market price available at the time of the transaction. Short selling was not permitted.

The platform allowed participants to continuously monitor price movements, portfolio composition, portfolio value, and performance data. In accordance with the experimental protocol, the rankings were available during the trading session.

3. Permitted Activities and Restrictions

Participants could buy eligible stocks, sell securities held in their portfolios, hold cash, or maintain positions without trading. They could place as many orders as they wished during the trading session, provided they complied with the platform’s rules.

The constraints were as follows: only CAC 40 securities were tradable, transactions were executed at market price, short selling was prohibited, and each participant managed only their own portfolio.

4. Recorded Data

The platform recorded the transaction history and the successive states of the portfolio. These data were used to calculate the number of transactions, the average order size, the average level of cash held, the final value of the portfolio, and the variability of that value during the trading session.

## Appendix E

### Interview Guide

**Table A5:** An overview of the semi-structured interview guide, including the main topics discussed, their objectives, and sample interview questions.

Topic Discussed	Objective	Sample Questions
General Attitude Toward the Stock Market and Risk	To understand the participant’s familiarity with the stock market, their attitude toward risk, and their mindset before the simulation	What is your attitude toward the stock market and risky financial decisions? What was your mindset before starting the simulation?
Emotional Profile During the Simulation	Identify the emotions experienced throughout the simulation and how they evolved from the beginning to the middle to the end	What emotions do you experience most often in this context? If you were to describe the simulation from an emotional perspective, what would it be like?
Positive Emotions	Understand the situations that bring you pleasure, satisfaction, confidence, or enthusiasm	What brings you pleasure or satisfaction in this type of simulation? In what situations do you feel confident or enthusiastic?
Negative Emotions	Identifying sources of fear, stress, tension, frustration, or discouragement	What causes the most fear, stress, or tension? How do you react to a loss or a bad decision?
The Link Between Emotions and Decisions	Understand how emotions influence decisions to buy, sell, or hold	Do you feel that your emotions influence your trading decisions? Have you ever regretted a decision made while in a particular emotional state?
Managing and Regulating Emotions	Identifying strategies used to control or reduce emotions during the simulation	When you feel your emotions rising, do you try to manage them? What strategies help you maintain some emotional distance?

## Appendix F

### Descriptive Statistics from the Semi-Structured Interviews

**Table A6:** The semi-structured interview guide used to explore participants’ attitudes toward the stock market, emotional experiences during the trading simulation, the influence of emotions on decision-making, and emotion regulation strategies, with the corresponding objectives and sample interview questions.

Participant	Duration	Number of Words	Number of Pages
P1	7 min and 40 s	1526	4
P2	18 min and 34 s	3524	14
P3	10 min and 25 s	1568	6
P4	12 min and 33 s	1793	6
P5	8 min and 39 s	1323	7
P6	11 min and 34 s	1830	7
P7	15 min and 1 s	2270	8
P8	12 min and 3 s	2042	8
P9	17 min and 33 s	2925	7
P10	25 min and 42 s	3798	9
P11	13 min and 3 s	1855	6
P12	19 min and 3 s	2452	7
P13	25 min and 25 s	4425	10
P14	21 min and 20 s	2114	7
P15	19 min and 21 s	2963	8
Average	15 min and 52 s	2427.2	7.6
Standard deviation	5 min and 41 s	915.6	2.3
Minimum	7 min and 40 s	1323	4
Maximum	25 min and 42 s	4425	14

## Appendix G

### Participant Characteristics

**Participant**	**Gender**	**Age**	**Prior Stock Market Experience**
P1	Male	20.	Yes, but limited to a previous classroom-based simulation
P2	Female	20.	No
P3	Female	19.	No
P4	Female	19.	No
P5	Female	33.	No
P6	Female	18.	No
P7	Female	19.	No
P8	Female	19.	No
P9	Male	20.	No
P10	Male	19.	No
P11	Female	24.	No
P12	Male	20.	No
P13	Female	20.	No
P14	Male	19.	No
P15	Male	19.	No
